# Mindfulness in Gastroenterology Training and Practice: A Personal Perspective

**DOI:** 10.2147/CEG.S278590

**Published:** 2020-11-04

**Authors:** Umakant Dave, Anjali Dave, Simon David Taylor-Robinson

**Affiliations:** 1 Department of Gastroenterology, Morriston Hospital, Swansea, Wales SA6 6NL, UK; 2 Department of Psychology, Birmingham University, Birmingham B15 2TT, UK; 3 Department of Surgery and Cancer, Imperial College London, St Mary’s Hospital Campus, London W2 1NY, UK

**Keywords:** mindfulness, gastroenterology, stress, meditation, wellbeing

## Abstract

**Background:**

Work-related stress is becoming an increasingly recognised occupational hazard that can have detrimental effects on the health of both patient and doctor. The practice of gastroenterology not only includes the demands of clinics and in-patient work faced by other medical specialities but also the additional burden of complex, and often high-risk, endoscopic interventions. Mindfulness, a secular form of meditation, can relieve stress, even if only practiced for a few minutes a day.

**Methods and Results:**

We present a personal perspective of the burnout experienced in stressful gastroenterology careers and the personal use of mindfulness in the daily routine to provide a source of calm when surrounded by many different pressures. We review some of the literature exploring the role of mindfulness in clinical practice with an emphasis on gastroenterology. While the practice of mindfulness is not designed to obviate immediacy and quick decisions in a rapidly changing clinical environment, it has been held widely useful to mitigate the stress involved in making those decisions.

**Conclusion:**

Practicing mindfulness, meditation and mindful living offers many advantages to gastroenterologists’ wellbeing as well improved patient care. We advocate its teaching to both gastroenterology trainees and consultants who are not familiar with the technique.

## Introduction

Mindfulness developed from Eastern religions, but its practice as meditation with health benefits has not reached the Western mainstream as much as yoga.

The psychological issues that accompany busy medical careers are often underplayed or completely neglected, but the stress involved may be both short and longer term in nature. Keswani and colleagues showed that most gastroenterologists in the United States experienced moderate levels of burnout, while junior gastroenterologists (those with three or fewer years of experience) had higher levels of stress that was related to endoscopic practice than senior gastroenterologists, regardless of the interventional procedures involved.^1^ While there has been an increasing trend to acknowledge workplace stress in an ever overstretched and resource-poor clinical environment, ways of effectively mitigating this have been lacking.

With stressful decision-making, the immediate problems of prioritising time can lead to anxiety. This can give rise to disturbed sleep patterns, tiredness the next day and a hang-over effect which further compound matters.

Mindfulness is distinct from other potential stress management techniques, such as going on vacation or watching television, both of which provide useful breaks from work. Problems do not go away when one returns from vacation and burnout levels usually return to pre-vacation levels 4 weeks later.^2^ There is no escaping from stress in life, especially working in clinical environments. However, instead of running away, an alternative approach is to change the way that we generate and relate to stress. Mindfulness allows practitioners to understand the generation of stress, accept the current situation, and recognise their automatic thought or behaviour patterns. These skills allow them to respond appropriately to a given situation. Mindfulness is not a way out of daily troubles, but instead, it is a “way in” by accepting and appropriately responding to them.

We present a personal perspective of mindfulness as a daily meditative tool for coping with busy medical practices and explore the possibility of its use in routine training programmes within the health service.

## Background of the Practice of Mindfulness

There has been an explosion of literature about the calming benefits of mindfulness over the last 20 years with an estimated 10% of the US population practicing meditation on a regular basis.^3^ However, despite mindfulness’s use in general society, there has been virtually no mindfulness education in gastroenterology training programmes in the United Kingdom, or in the majority of training programmes worldwide. We believe there are many reasons why a practicing gastroenterologist should understand and consider the practice of mindfulness, which has not only personal benefits, but also beneficial effects on medical teams and patients alike:

1. There is a high rate of stress, mental health problems and burnout in gastroenterologists; the practice of mindfulness has been shown to improve wellbeing in such situations.
2. There is some evidence that mindfulness training improves cognitive function and performance of surgeons. One can extrapolate therefore, that gastroenterologists performing complex endoscopic interventions may also benefit.
3. Mindfulness training improves communication, leading to improved team-work and patient satisfaction. Mindfulness training has been suggested to help in reducing medical errors.
4. There is evidence that mindfulness training helps patients with many gastrointestinal (GI) problems, such as irritable bowel syndrome (IBS), inflammatory bowel disease (IBD), and GI cancer. Personal knowledge of mindfulness may help the supervising gastroenterologist to refer appropriate patients for mindfulness training.

We will now explore these issues in detail:

## Reduction in Stress, Burnout and Improvement in Wellbeing

Burnout is defined in the 11th revision of the International Classification of Diseases (ICD-11) as a “syndrome conceptualized as resulting from chronic workplace stress that has not been successfully managed”. It is characterized by three dimensions: feelings of energy depletion or exhaustion; increased mental distance from one’s job, or feelings of negativism or cynicism related to one’s job; and reduced professional efficacy^4^.Burnout has been noted in busy professionals including police officers, doctors and other healthcare professionals.^5^ Burnout in the context of modern medical practice is now commonplace. For example, prevalence of burnout in gastroenterology trainees within the East of England Deanery was 35%.^6^ In a British Medical Association (BMA) survey on the subject, 40% of respondents reported currently suffering from a broader range of psychological and emotional conditions, as well as burnout.^7^

Burnout contributes to medical errors, decreased patient satisfaction, a decrease in doctor participation within the hospital environment, and losses to the physician workforce through early retirement.^8^ Endoscopic procedures leading to procedural stresses (complications, missed diagnosis, failure and misdiagnosis) liken the practice of gastroenterology to a surgery practice, in contrast with non-procedure-based internal medicine subspecialties. Dispositional mindfulness, which in a US study was defined as the “trainable ability to pay attention to inner thoughts, emotions, and experiences in a non-reactive way”, has been shown to have a calming effect on perceived stress and burnout amongst both senior healthcare workers and trainees.^9^

Another US study amongst surgical trainees found that the practice of mindfulness conveyed a reduced incidence of workplace burnout and distress symptoms.^10^ Similarly, work by Dobkin and colleagues showed significant reductions in stress and burnout through the practice of mindfulness and with Mindfulness Based Stress Reduction (MBSR) training. A decrease in emotional exhaustion was correlated with the ability to act with awareness and have less judgmental attitudes; both of which are facets of mindfulness.^11^

While a large number of studies show significant improvement in wellbeing and performance of doctors and other healthcare professionals following MBSR, many studies are of low quality. A systematic review concluded that mindfulness could have a positive impact on health professionals’ ability to deal with stress. However, certain methodological limitations of the training were also highlighted.^12^ Nevertheless, the role of mindfulness in addressing healthcare workers’ burnout is perhaps promising. After several weeks of meditation-based techniques, 23 of the 34 published studies reported an improvement on measured burnout criteria.^13^

In support of the utility of mindfulness in the context of burnout, in a cross-sectional study from the United States, surgical trainees with burnout and high stress were found to be at higher risk for depression and suicidal ideation. The practice of mindfulness was associated with lower risk of burnout and distress symptoms, supporting the use of mindfulness training in promoting resilience during surgical training programmes.^10^

## Improvement in Cognitive Functions and Performance

In a randomised controlled study of first-year surgical residents, motor performance and aspects of executive function, including working memory capacity, improved in the MBSR group compared with the control group.^14^ In this study, Lebares and colleagues showed potential benefits of mindfulness training to residents’ well-being by demonstrating twice as much increase in mindfulness scores in the modified MBSR (modMBSR) arm as in the control arm, whereas stress and depressive symptoms scores increased twice as much in the control group as in the modMBSR arm.^14^

A systematic review of 153 studies suggests that mindfulness can potentially reduce mental health issues (such as stress), enhance well-being-related outcomes (such as job satisfaction) and improve aspects of job performance (such as compassion and empathy).^15^

## Reduction in Medical Errors and Improvement in Patient Satisfaction

Patients’ perceptions of clinical encounters suggest that patient-centred care improved after MBSR.^11^ In a randomised controlled trial of 124 psychiatric inpatients managed by 18 psychology residents, Grepmair and colleagues showed that patients of interns who received mindfulness training did significantly better than those patients treated by interns who did not receive mindfulness training.^16^ One study found that doctors who were more mindful with their patients were more upbeat, better listeners and showed more empathy while remaining efficient in their daily work tasks.^17^

## Mindfulness for Patients

Aside from the benefits of mindfulness for healthcare practitioners, exploring mindfulness for patients is something that needs consideration. The impact of a diagnosis is stressful, particularly those carrying with them potentially lifelong morbidity, such as ulcerative colitis or Crohn’s disease. The impact of cancer is well known, but often the psychological aspects of a malignant condition are not adequately addressed in a busy healthcare service. There is a paucity of well-defined large scale randomized-controlled trials on holistic treatments for functional GI problems like IBS, but group mindfulness sessions can introduce the concept to patients, who, if they so choose, can incorporate this technique into their daily private routine.

A meta-analysis showed that mindfulness-based interventions may provide benefit in functional gastrointestinal disorders. Pooled mean effect sizes were statistically significant for IBS severity (0.59, 95% CI 0.33 to 0.86) and quality of life (0.56, 95% CI 0.47 to 0.79).^18^ Mindfulness interventions for patients with IBD may be a supplemental treatment option to improve quality of life and distress in this population.^19^ Furthermore, mindfulness is being increasingly considered a standard therapy in psycho-oncology.^20^

## Personal Perspective

A recently-published review article states that doctors are most likely to become mentally ill when they feel isolated or unable to do their job.^21^ The current situation is extreme, as a result of the COVID-19 crisis. As the reality of near-total societal shutdown and personal risks to front-line workers in healthcare dawns, new layers of personal apprehension and uncertainty compound commonplace anxieties, as well as increased feelings of isolation. Personally, we have, as individuals, found that the practice of mindfulness has strengthened our resilience to stress, with a lower incidence of experiencing distress symptoms during times when practicing the technique regularly. We have felt that decision making has improved, efficiency has been maintained in fast-moving clinical environments and relationships with colleagues have been much more constructive, all leading to better patient service. These are of course our personal observations (without any objective measures) and may not apply to everyone.

Breathing meditation, body scan meditation and open awareness meditation help to improve attention and reduce the power of negative thoughts. Loving-kindness meditation entails the active generation of friendliness and goodwill towards the self and others, including people with whom there may be a difficult relationship. MBSR training incorporates training in all these meditations in a secular fashion and also encompasses skills training to incorporate mindful living to daily activities.

One of the authors (UD) joined a MBSR course 10 years ago, following a recommendation by one of his patients who had found MBSR helped her with bowel symptoms and stress. Since then UD has been practising meditation regularly and has attended teacher training retreats at Bangor University. UD has run many short “Introduction to Mindfulness” sessions and “Mindfulness and Wellbeing” study days. UD has also run a “Debrief and Wellbeing” weekly session for hospital staff during the height of the COVID-19 pandemic, which included supportive conversation and group meditation. Admitting vulnerabilities and sharing stories of kindness has helped the group deal with crises.

## Some Practical Tips for Mindfulness Practice

- Focus on the point where you feel your breath most and observe your emotions and body’s sensations without judgment before/during the stressful time.
- During any form of exercise, bring your attention to your body’s sensations.
- Download a meditation app (there are many free apps available).
- Consider joining a MBSR course.

## Training Programmes

MBSR is an eight-week evidence-based programme that offers secular, intensive mindfulness training. It is a practically-based approach, which focuses on intention, attention and attitude. Improved attention allows people to cultivate awareness and thereby enables a greater ability to make considered decisions when moving from reaction to response. Developed at the University of Massachusetts Medical Centre in the 1970s by Professor Jon Kabat-Zinn, MBSR uses a combination of formal practices (mindfulness meditation, body awareness, movement practice), and informal practices (incorporation of principles in daily activities). The MBSR program is a workshop taught by certified trainers that entails weekly group practice (2.5-hour classes), a one-day retreat (7-hour mindfulness practice) between sessions six and seven, homework (45 minutes daily), and instruction in three formal techniques: mindfulness meditation, body scanning and simple yoga postures.^22^

## Discussion

William James wrote in 1961

The faculty of voluntarily bringing back a wandering attention, over and over again, is the very root of judgment, character, and will. No one is compos sui [master of himself] if he have it not. An education which should improve this faculty would be the education par excellence.^23^

Furthermore, Killingsworth and Gilbert added;

people’s minds wander frequently. This ‘mind wandering’ appears to be the brain’s default mode. This prominent ability may have an emotional cost. People are less happy when their minds are wandering.^24^

Meditation has proven difficult to define as it covers a wide range of dissimilar practices in different traditions. Walsh and Shapiro refer to meditation as “a family of self-regulation practices that focus on training attention and awareness in order to bring mental processes under greater voluntary control”.^25^ The technique thereby fosters general mental well-being and as a consequence, the “development of specific capacities, such as calmness, clarity and concentration”.^25^ Mindfulness is the psychological process of purposely bringing attention to experiences occurring in the present moment without judgment, which develops through the practice of meditation and other training.

There are limited cohesive, overarching attempts to describe the various psychobiological processes that meditation sets in motion and the scientific basis of its effect. A review by Reive included studies looking at the psychobiological aspects of MBSR.^26^ It demonstrated that following MBSR, the prefrontal cortex, responsible for integration and cognition, exhibited decreased connectivity with the amygdala. The amygdala is responsible for the “fight or flight” stress response, perhaps tempering its reactivity through learned non-judgmental, in-the-moment awareness. The evaluated studies support the premise that MBSR can decrease systolic and diastolic blood pressure but have not yet demonstrated an effect on heart rate variability or respiratory measures. The examined MBSR literature shows preliminary support for a reduction in cortisol, CRP, decreased natural killer cell activity, decreased NF-kB (a protein complex that controls transcription of DNA) and increased or buffered declines of CD4^+^ T cell counts (Th1 and Th2) which facilitates a return to allostasis. By practicing MBSR techniques, improvements can be seen in the brain regions responsible for perception, memory and response flexibility occur which translates to decreased stress reactivity. This modified reaction by the brain appears to influence the body’s stress response, resulting in improvements of the autonomic, immune, inflammatory and endocrine responses.^26^

Mindfulness has the potential to support wellbeing in healthcare professionals and our patients, but individuals and providers need to be aware of its downsides. It should be noted that MBSR is demanding in terms of individual commitment and organisational resources. Currently, there is no professional or statutory registration required to teach mindfulness-based interventions such as MBSR and MBCT (Mindfulness Based Cognitive Therapy) and no regulatory body which oversees the training of mindfulness teachers. Further, qualitative research on mindfulness meditation shows that it may increase the awareness of difficult feelings and exacerbate psychological problems.^27^ Therefore, rather than being touted as a panacea, we must understand for whom and under what circumstances it works and when it may be contraindicated. Having first-hand knowledge of mindfulness in our own lives allows an insight into who may or may not benefit from the technique as far as patients are concerned.

The Association of Anaesthetists of Great Britain and Ireland summarises the role of mindfulness succinctly;

mindfulness will not make your problems disappear, but it will help you break patterns of unhelpful behaviour, such as being self-critical or not prioritising your own wellbeing. It will help you to stop wanting things to be different and instead help you accept things the way they are. Being mindful allows you to better observe patients’ responses while remaining focused and aware of tasks and surroundings. It decreases errors and improves patient safety. It gives you space to ‘be’ and will help you respond to stressful situations more calmly and with less anxiety.^28^

While conventional treatment algorithms are absolutely paramount for our patients, a holistic approach employing mindfulness may be helpful in those who find the impact of bad news disturbing or stressful. Equally, the stresses of modern-day medicine might be alleviated in busy healthcare professionals by some knowledge of this meditative art, even if it is not practiced on a regular basis. Finally, it is critical to emphasize that one can derive real benefit from meditation without an extensive time commitment. As little as 13 minutes a day, if practiced daily, is beneficial to wellbeing.^29^
